# Coupling Infusion and Gyration for the Nanoscale Assembly of Functional Polymer Nanofibers Integrated with Genetically Engineered Proteins

**DOI:** 10.1002/marc.201500174

**Published:** 2015-06-01

**Authors:** Siqi Zhang, Banu Taktak Karaca, Sarah Kay VanOosten, Esra Yuca, Suntharavathanan Mahalingam, Mohan Edirisinghe, Candan Tamerler

**Affiliations:** ^1^ Department of Mechanical EngineeringUniversity College LondonTorrington PlaceLondonWC1E 7JEUK; ^2^ Bioengineering Research Center (BERC)Department of Mechanical EngineeringUniversity of Kansas (KU)LawrenceKS66045USA

**Keywords:** genetically engineered proteins, gyration, infusion, nanofibers, peptides

## Abstract

Nanofibers featuring functional nanoassemblies show great promise as enabling constituents for a diverse range of applications in areas such as tissue engineering, sensing, optoelectronics, and nanophotonics due to their controlled organization and architecture. An infusion gyration method is reported that enables the production of nanofibers with inherent biological functions by simply adjusting the flow rate of a polymer solution. Sufficient polymer chain entanglement is obtained at Berry number > 1.6 to make bead‐free fibers integrated with gold nanoparticles and proteins, in the diameter range of 117–216 nm. Integration of gold nanoparticles into the nanofiber assembly is followed using a gold‐binding peptide tag genetically conjugated to red fluorescence protein (DsRed). Fluorescence microscopy analysis corroborated with Fourier transform infrared spectroscopy (FTIR) data confirms the integration of the engineered red fluorescence protein with the nanofibers. The gold nanoparticle decorated nanofibers having red fluorescence protein as an integral part keep their biological functionality including copper‐induced fluorescence quenching of the DsRed protein due to its selective Cu^+2^ binding. Thus, coupling the infusion gyration method in this way offers a simple nanoscale assembly approach to integrate a diverse repertoire of protein functionalities into nanofibers to generate biohybrid materials for imaging, sensing, and biomaterial applications.

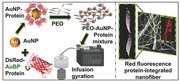

## 1 Introduction

Inorganic‐binding peptides have attracted tremendous interest in the last decade, ranging from assembly of nanoparticles^1^, ^2^ to oriented immobilization of proteins,^3^, ^4^, ^5^ synthesis of inorganics,^6^, ^7^ and biofunctionalization of surfaces.^8^, ^9^, ^10^, ^11^, ^12^ Solid‐binding peptides have been shown to control the organic–inorganic materials interface in different applications, but their integration to design tunable, functional nanofibers has not been investigated. This peptide‐based nanoassembly process can be regulated at the molecular level to construct a self‐organized architecture and establish an ordered nanostructure.

Over the past decade, a variety of techniques have been used to produce nanofibers and among these pressurized gyration process, invented in 2013, has generated exceptional interest for the mass production of functionalized products including fibers and microbubbles.^13^, ^14^ However, one drawback is that it does not allow control of fluid flow through the fiber generating orifices where the infusion rate of polymer solution influences fiber size and distribution, and the morphology of the spun fiber. Here, we have created a novel method, infusion gyration, which does not require external pressure, but rather, relies on controlling the infusion rate of the polymer solution. This allows to generate nanofibers that are integrated with nanoassemblies, which in this work feature gold nanoparticles and inorganic‐binding peptides. Namely, we utilized the well‐characterized gold‐binding dodecapeptide, Au‐BP2,^15^ for the fiber formation process. As a means to easily trace the integration of Au‐BP2 into the nanofibers, we genetically conjugated the gold‐binding peptide to a biomarker protein, i.e., red fluorescence (DsRed). Additionally, the protein goes through reversible fluorescence quenching upon copper ion binding. Thus, we show that the biohybrid nanofibers could be further exploited to advance protein‐integrated functional materials for biofabrication.

## 2 Experimental Section

### 2.1 Materials

Polyethylene oxide (PEO, powder, molecular weight ≈ 200 000 g mol^−1^) and gold nanoparticle solution (analytical grade, average particle size ≈10 nm) were purchased from Sigma–Aldrich (Poole, UK). All reagents were used without further purification. Isopropylthiogalactopyranoside (IPTG) was purchased from Sigma–Aldrich (Milwaukee, WI, USA). Amylose resin for column chromatography was purchased from New England Biolabs (Ipswich, MA, USA). The Instant Blue coomassie based staining solution was procured from Expedeon Inc. (San Diego, CA, USA). All buffers were filtered and degassed before using. Details on expression and purification of red fluorescent gold‐binding fusion proteins are given in Supporting Information and the genetic construction of the plasmid was described in our previous work.^16^

### 2.2 Preparation of Polymer and Engineered Protein Solution Mixture

The polymer solution was prepared in an air‐tight bottle using deionized water as solvent to dissolve the PEO powder under magnetic stirring for 24 h at ambient temperature (20 °C). Solutions with various concentrations of PEO were prepared, however, as explained below, only the 10 wt% of PEO solution was chosen to integrate with the protein–nanoparticle assemblies. Phosphate buffer saline (PBS) solution with a pH ≈ 7.4 was prepared at ambient temperature and added to the purified DsRed‐AuBP2‐engineered protein (molecular weight ≈ 30 kDa) using a micropipette to achieve a working stock solution of 50 × 10^−6^ m. The gold nanoparticle solution was added to the gently shaken protein solution. 40 g of PEO solution was taken in an air‐tight bottle and 0.4 mL of the gold nanoparticle–protein mixture was added while sonicating in a water bath using an ultrasound sonifier (Branson sonifier 250) at a power output of 60% for 15 min. This prevented aggregation of the functionalized gold nanoparticles in the polymer solution.

### 2.3 Infusion Gyration

**Figure 1:**
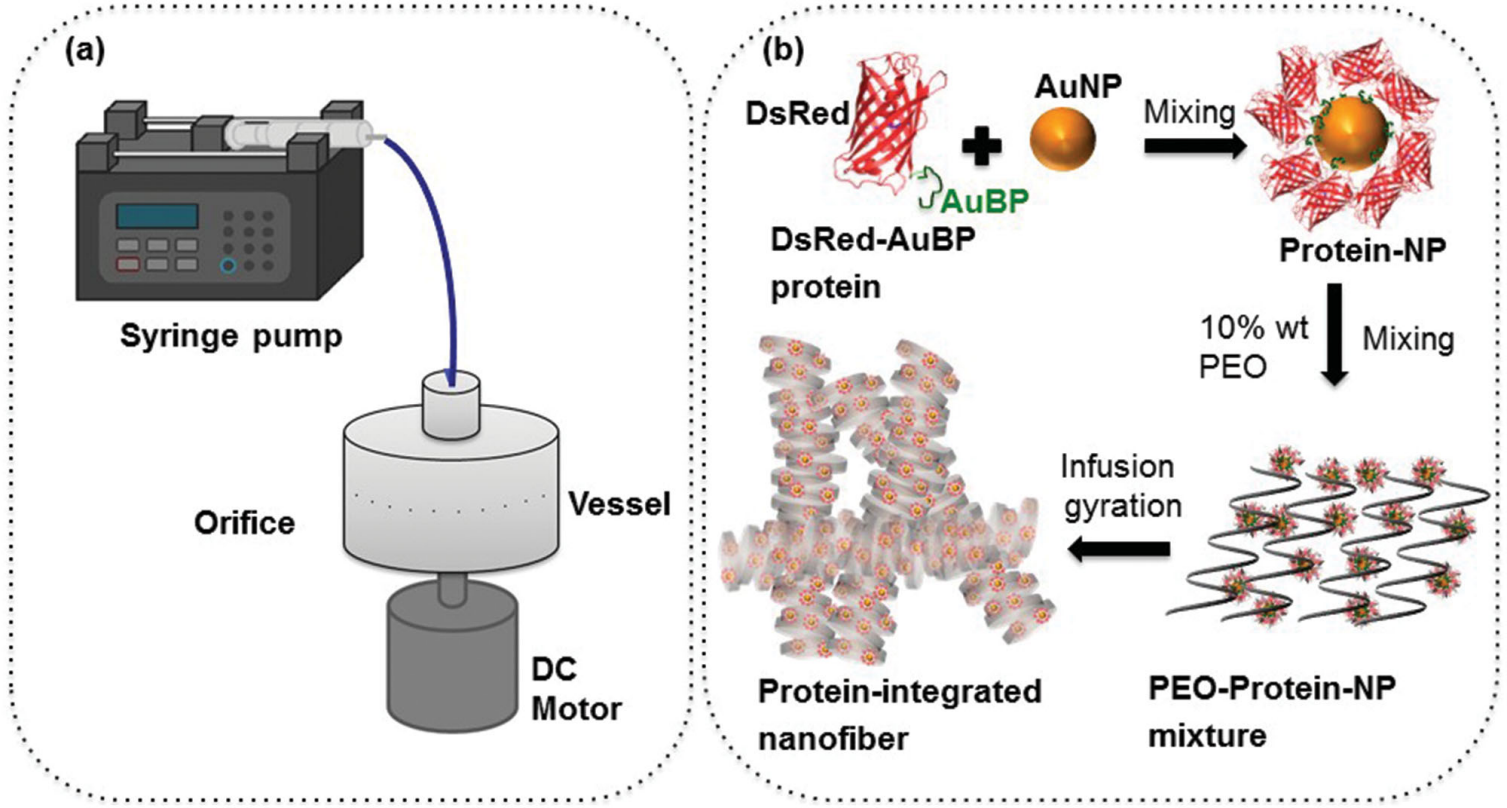
a) Basic simple experimental set up of the infusion gyration process. b) Schematic illustration of the formation of engineered Ds‐Red fluorescence protein‐integrated nanofibers, integration may be non‐uniform along the fiber surface.

The system consists of a rotary aluminum cylindrical vessel containing 20 small round orifices on the face (Figure 1a) much like in our work with pressurized gyration.^13^, ^14^ The dimensions of vessel and orifices are 60 mm in diameter with a height of 25 mm, and 0.5 mm in diameter located at the same vessel height, respectively. One end of the vessel was joined to a syringe pump through a rotary joint, which can control flow into the vessel. The bottom end of the vessel was connected to a DC motor, which can produce variable speeds up to 36 000 rpm. In order to investigate the nanofiber size and size distribution under different conditions, the polymer solution was spun at six different flow rates (5000, 4000, 3000, 2000, 1000, and 500 μL min^−1^) under a constant rotating speed (36 000 rpm) at the ambient temperature and relative humidity (≈42%). Protected by a transparent plastic container, the system allows convenient collection of the polymer fibers on stationary aluminum foil sheets within the container. A schematic illustration of the different steps in the entire process is shown in Figure 1b.

### 2.4 Fiber Characterization

Nanofibers produced were studied using field‐emission scanning electron microscopy (FE‐SEM). Samples were coated with gold using a sputtering machine (sputter time ≈75 s) before loading to the microscope. High‐ and low‐magnification images were acquired at randomly selected positions (>20) within a sample. About 150 measurements were made at random locations to plot the fiber diameter distribution using ImageJ software. Additionally, the as‐produced fibers were characterized using a fluorescence microscope to verify the integration of the DsRed fluorescence proteins expressed with AuBP peptides in the nanofibers.

### 2.5 Fourier Transform Infrared Spectroscopy

The infrared spectra of fibers were recorded on a PerkinElmer Spectrum‐400 Fourier transform infrared spectroscopy (FTIR) spectrometer at the ambient temperature between 4000 and 650 cm^−1^ with a resolution of 4 cm^−1^. To obtain reasonable signal‐to‐noise ratio, the average of 20 scans was taken. Samples were analyzed directly by single‐bounce diamond ATR.

### 2.6 Localized Surface Plasmon Resonance Spectroscopy

The effect of proteins on the plasmon excitation wavelength for gold nanoparticles (AuNPs) was analyzed by measuring the light absorbance of AuNPs in the absence and the presence of protein‐conjugated AuNPs in the nanofiber using a Cytation 3 Imaging Multi‐Mode Plate Reader (BioTek Instruments, Inc., Vermont, USA). Each spectrum is an average of three individual samples recorded twice.

### 2.7 Copper‐Binding Assay

Different concentrations of copper were added to the DsRed‐AuBP2c protein fiber solution. The fluorescence intensity was measured (Varian Cary Eclipse Fluorescence Reader) by excitation of the samples at 556 nm and emissions were recorded at 590 nm.

## 3 Results and Discussion

**Figure 2:**
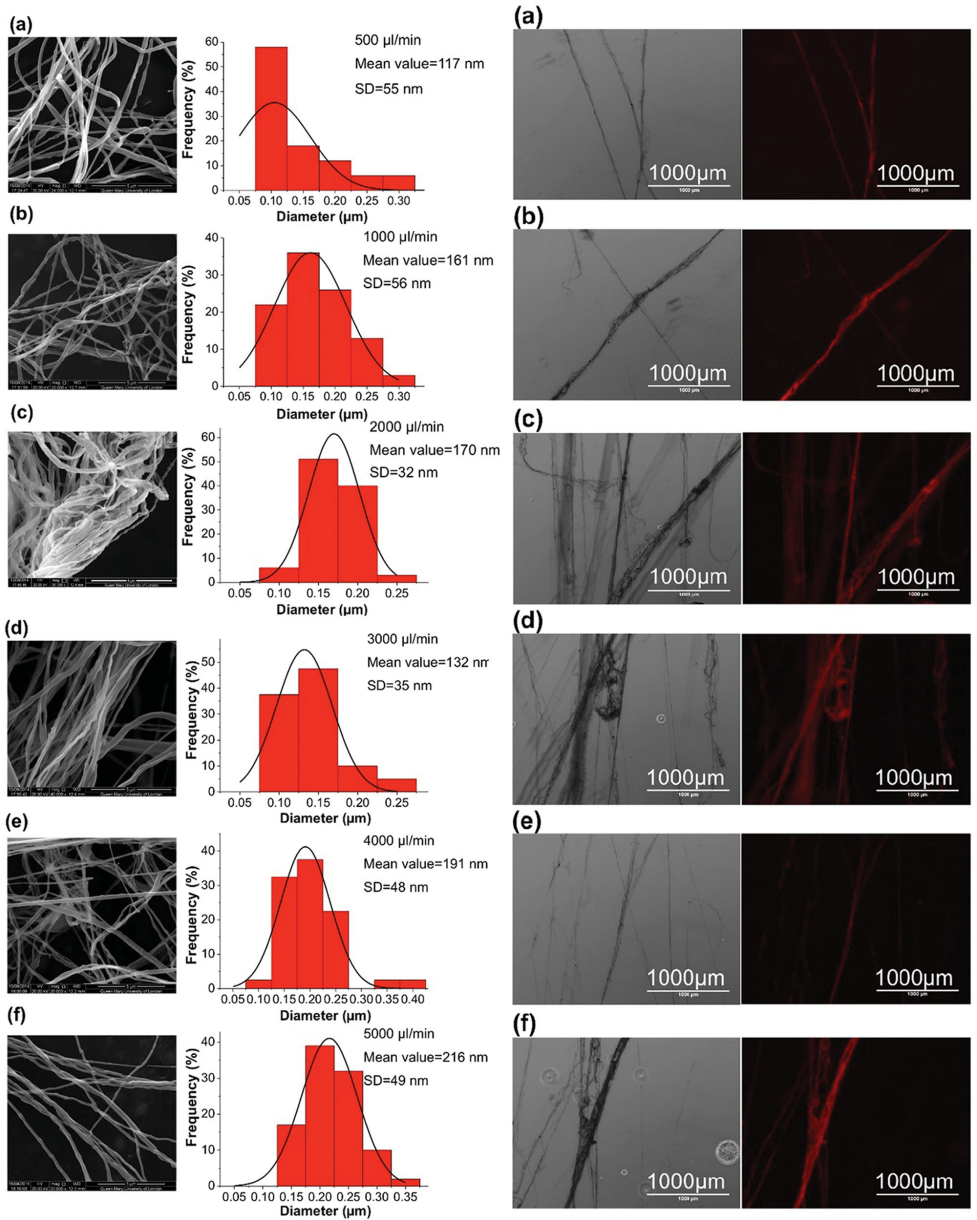
Typical FE‐SEM images, diameter distributions and fluorescence microscopy images of the protein‐integrated fibers produced at flow rate a) 500 μL min^−1^, b) 1000 μL min^−1^, c) 2000 μL min^−1^, d) 3000 μL min^−1^, e) 4000 μL min^−1^, f) 5000 μL min^−1^, all at a fixed rotating speed 36 000 rpm.

Detailed information on protein production and their gold‐binding characteristics are given in Supporting Information. Figure 2 provides details regarding the fiber size (diameter) and size distribution of the protein‐integrated nanofibers produced. These ranged from 117 to 216 nm in average diameter for the six different flow rates studied. At the lowest flow rate, 500 μL min^−1^, the mean fiber size diameter was 117 nm. When the flow rate was doubled to 1000 μL min^−1^ the mean fiber size was 161 nm. Surprisingly, the flow rate of 3000 μL min^−1^ resulted in a reduced mean fiber size of 132 nm and this is explained below. The initial size increasing trend continued at higher flow rates, and, at 4000 μL min^−1^ size was 190 nm, and at 5000 μL min size was 216 nm. The polydispersities of the fiber size distributions were 47%, 32%, 19%, 26%, 25%, and 23% for flow rate values of 500, 1000, 2000, 3000, 4000, and 5000 μL min^−1^, respectively, with the rotating speed remaining constant at 36 000 rpm. The morphology of nanofibers revealed bead‐free, continuous, uniform structures. Single‐strand pore‐free fibers were bundled together and it was also possible to form well‐aligned structures due to the high stretching force experienced during gyration.

Compared to pressurized gyration,^13^ the ability to use higher flow rates allows increased hydrostatic pressure, which was kept constant for flow rates at a fixed rotating speed by ensuring continuous flow. But the hydrostatic pressure is much lower than the centrifugal force at the orifice.^17^ This allows the destabilizing centrifugal force and the withholding surface tension force to determine the final size and size distribution of the fibers. Moreover, the flow rate regulates the volume of material and the mass transfer across the orifice. The overall increase of fiber diameter with increasing flow rate is attributed to this phenomenon. The drop in fiber diameter observed at 3000 μL min^−1^, and subsequent increase at higher flow rates may be attributed to the balance of solvent evaporation and change in the volume of material at the orifice. At lower flow rates, the solvent has enough time to evaporate, allowing the polymer jet more time to stretch, which results in the formation of finer fibers. At higher flow rates, the solvent does not have sufficient time to evaporate before reaching collection, therefore, a coarser fiber is formed.^18^, ^19^ Additionally, in infusion gyration, the volume and shape of the polymer droplets vary for different flow rates at the orifice, which could cause differences in fiber size and size distribution. Finally, during spinning, the traveling polymer jet experiences aerodynamic forces that may impact the stretching of the jet and, thereby further alter the final size and size distribution.^20^

In fiber‐forming processes, viscosity and concentration of the polymer solution influence the resulting fiber size and fiber morphology.^13^, ^21^, ^22^ In a typical pressurized gyration process, these properties also affect polymer chain entanglement, a prerequisite to the formation of nanofibers.^13^ To verify that this is also the case in infusion gyration, we adopted analysis using the Berry number (Be), a dimensionless index used to control and indicate the fiber size. Be = [η]*C*, and [η] represents the intrinsic viscosity (0.32 dL g^−1^ for PEO), and *C* is the concentration.

A clear‐cut relationship is evident (Table 1) where, as the concentration was increased, viscosity (η) increased gradually until a specific value was reached, after which the viscosity increased dramatically, giving, *η* = 0.92*C* ^2.73^. In dilute polymer solutions, Be < 1.6, and nanofibers could not be formed due to insufficient polymer chain entanglement. When 1.6 < Be < 3.2 nanofibers formed only at a rotating speed of 36 000 rpm, primarily due to the increased time constant of forces acting upon the polymer solution and the increased viscosity of the polymer solution. Thus, a minimum rotating speed of 36 000 rpm was required to initiate fiber formation. For Be numbers between 3.2 and 4.8, a sufficient amount of chain overlap and entanglement allowed the formation of nanofibers. However, when Be > 4.8, a much thicker fiber resulted. Therefore, a 10 wt% PEO polymer solution was selected for integration of AuNPs functionalized with DsRed‐AuBP2 to the nanofibers.

**Table 1:** Polymer concentration, viscosity, and Berry number for PEO solutions at ambient temperature

Polymer concentration [wt%]	Viscositya) [mPa s]	Surface tensionb) [mN m^−1^]	Berry number (Be)
5	75 ± 4	50 ± 1	1.6
10	390 ± 23	51 ± 1	3.2
15	2200 ± 75	52 ± 2	4.8
21	3000 ± 86	57 ± 2	6.7

^a)^Brookfield viscometer used;

^b)^Kruss tensiometer used.

Protein‐integrated PEO nanofiber yield steadily increases on increasing the infusion rate from 500 μL min^−1^ (0.02 kg h^−1^) until 3000 μL min^−1^ (0.18 kg h^−1^), after which the yield increased dramatically by nearly an order of magnitude to 1.45 kg h^−1^ at an infusion rate of 5000 μL min^−1^. However, yields obtained for the infusion gyration method was lower than that achieved from pressurized gyration. This may be due to the absence of blowing in the former, but it should be noted that the yields are still greater than those achieved through conventional centrifugal spinning or electrospinning.^13^

**Figure 3:**
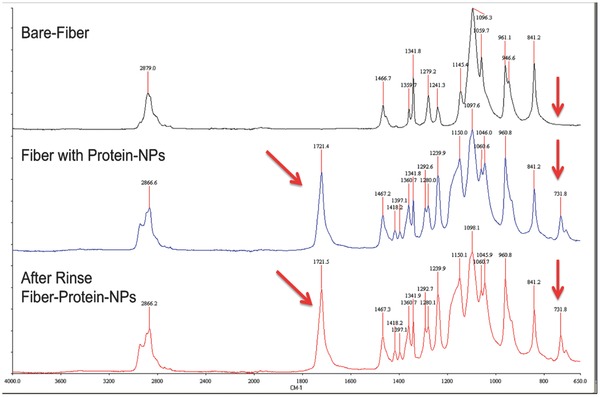
FTIR spectra of the different nanofiber samples. X‐axis is wavenumber (cm^−1^).

Concerning the images provided in Figure 2, the left panels are bright‐field and the right panels are the corresponding fluorescence images. These reveal the smooth structure of the nanofibers and regardless of the flow rate variation, they indicate that the integration of engineered protein with gold‐binding peptides within the nanofibers is possible. FTIR analysis conducted on PEO and biohybrid (PEO/protein) fibers confirmed the presence of the protein. Figure 3 depicts that characteristic peaks of PEO observed at 2900 cm^−1^ (methylene group CH_2_ molecular stretching), and at 1100 cm^−1^ and 960 cm^−1^ (C—O—C group stretching).^23^, ^24^, ^25^ A change in bandwidth for the absorption centered around 2880 cm^−1^ also occurred and changed further with protein integration. Engineered proteins in the PEO/protein nanofibers resulted in an FTIR peak at 1720 cm^−1^, representing the characteristic amide bonds of protein.^25^ After washing the PEO/protein nanofibers with PBS, the same FTIR peak was still observed.^26^ The maintained carbonyl peak suggests that the protein remained bound to the surface of the AuNPs, which were integrated in the PEO nanofibers, even following a washing step. This is remarkable protection of the water‐soluble PEO polymer and is further discussed below.

**Figure 4:**
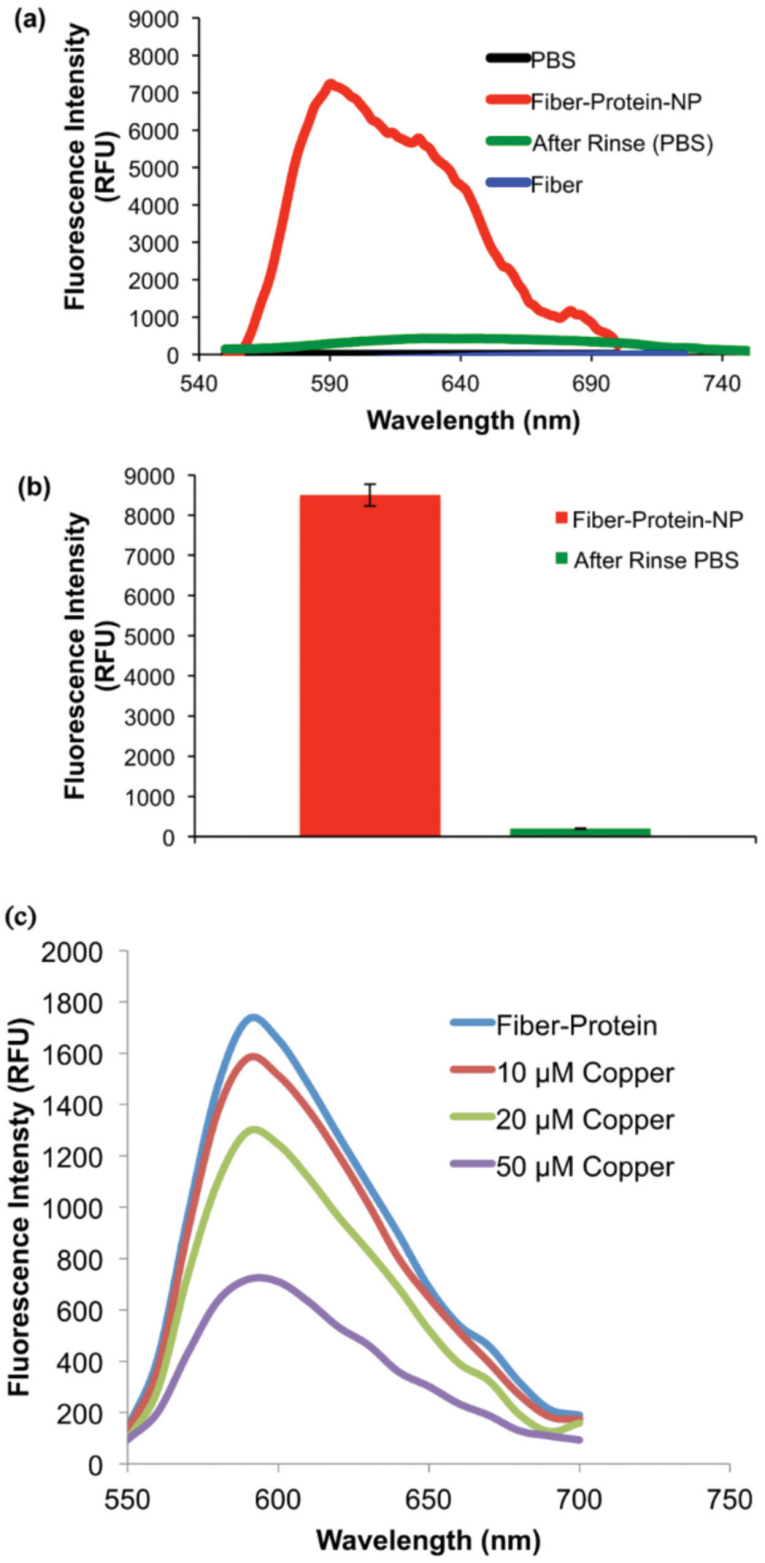
Optical characteristics of PEO and PEO/protein nanofibers measured by fluorescence spectrophotometry. a) Nanofibers containing red fluorescence protein in PBS buffer. b) The removal of the PEO/protein nanofibers from PBS buffer diminishes fluorescence intensity demonstrating no significant protein leakage into the solution. c)Titration of DsRed‐AuBP2‐integrated nanofiber with Cu^2+^. Emission spectra were obtained by excitation at 558 nm in the presence of 10 × 10^−6^, 20 × 10^−6^, 50 × 10^−6^ m Cu^2+^.

Many efforts have been made to decorate nanofibers with metallic nanoparticles.^27^ Here, genetically engineered proteins were used to stabilize AuNPs and direct their spatial order into 1D particle assemblies (Figures S3 and S4, Supporting Information). Integrating discrete AuNPs into the hybrid nanofibers may significantly alter their inherent optical properties, which were evaluated by UV–Vis and fluorescence spectrophotometry. We observed that integration of AuNPs onto the surface of the fibers had a significant effect, when compared to non‐protein‐integrated fibers. Hybrid nanofibers in PBS buffer exhibited a strong fluorescence emission band at wavelength 570 nm (Figure 4a). Interestingly, protein‐based nanofibers did not dissolve in PBS buffer and did not show any leakage of protein to the buffer. When nanofibers were removed from the PBS buffer, the fluorescence intensity of PBS buffer alone was completely mitigated confirming that the red fluorescent protein did not leak into PBS buffer (Figure 4b).

This might be related to peptide‐enabled self‐organization of fusion protein onto nanoparticles, which may be stabilizing the polymer complex. The resulting protein with gold‐binding peptide tag (DsRed‐AuBP2) is hypothesized to blend into fiber formation by providing self‐organized biomolecular–inorganic surface interactions to decorate gold nanoparticles along the nanofibers. The functionalized‐gold nanoparticles with inorganic‐binding peptide potentially provide both electrostatic and steric interactions to stabilize nanofiber formed by the polymer PEO and consequently this may result in different phase behavior of the hybrid polymer.

Additionally, biohybrid nanomats composed of nanofibers were analyzed in both dry and wet conditions using a cell‐imaging multi‐mode reader and indicates that protein‐integrated samples did not dissolve in aqueous environments and that similar fluorescent images were obtained in both dry and wet forms (Figure S5, Supporting Information). Thus, protein‐based nanofibers did not show any leakage of protein into the PBS buffer, which was verified through a fluorescence analysis of the buffer solution after the nanofibers were removed. Interestingly, as expected, PEO nanofibers that were not integrated with proteins dissolved completely when exposed to an aqueous environment, suggesting a stabilizing role of the protein nanoassemblies on the nanofibers. Here, engineered DsRed‐AuBP2 proteins were utilized due to their low autofluorescent potential given that their excitation and emission wavelengths in the near‐far‐red region of the spectrum allow for a higher signal‐to‐noise ratio. However, the protection of the water‐soluble PEO polymer and the intact DsRed‐AuBP2 proteins in the nanofibrous structures may be correlated to phases present in the complex polymer system and this requires a more in‐depth study. It has been shown that phase separation can occur in the radial direction of fibers due to rapid solvent evaporation during spinning, thus a poly­mer‐rich phase occurs in the inner regions of the spun hybrid fibers whereas a secondary phase can be locked onto the outer surface of the drying liquid jet.^28^ Moreover, PEO can crystalize at the ambient temperature thus preventing the rearrangement of amorphous chains that might influence the formation of secondary structures on the surface of the nanofibers.^29^

Our previous studies^16^ found that significant quenching of the red fluorescence activity occurred in the presence of copper ions. Thus, we explored the potential of using the nanofibers for rapid monitoring for the presence of copper ions, a major component in heavy metal pollution. Different concentrations of copper were added to nanofiber samples in 100 μL of MOPS buffer and fluorescence readings were measured at 558 and 590 nm excitation and emission wavelengths, respectively. The nanofibers demonstrated quenching at different copper concentrations (Figure 4c). This promising result not only demonstrates the potential of protein‐integrated nanofibers to monitor the presence of copper ions in solution, but also establishes the potential impact that may be achieved through further tuning of the system to detect biological and chemical changes in the environment. Our results provide evidence that the red‐fluorescence activity of the engineered proteins embedded in the nanofibers maintain full functionally and can respond to various dynamic conditions.

## 4 Conclusion

An infusion gyration method that enabled the production of engineered protein‐integrated nanofibers has been invented. By adjusting the flow rate of the polymer solution, well‐aligned, smooth‐and bead‐free‐nanofibers were generated. FTIR analysis confirmed the presence of the protein, by revealing the characteristic peak of the amide bond in the protein structure in the nanofibers. The assembly of the engineered proteins bound to the gold nanoparticles along the fibers was easily tracked by exploiting the fluorescent activity of the coupled proteins in the nanoassembly. Gold nanoparticles chaperoned the bifunctional protein integration into the nanofibers. The nanofibers produced also become much less soluble in water, due to the incorporation of the protein‐coated gold nanoparticles. Copper‐induced fluorescence quenching of red fluorescence protein was observed when the fibers were exposed to an increased level of copper concentration. As the protein‐infused fibers were able to maintain their biological functionality, even in response to changes in buffer conditions, they can offer a fundamental platform in the design and fabrication of novel biomaterials.

## Supporting information

As a service to our authors and readers, this journal provides supporting information supplied by the authors. Such materials are peer reviewed and may be re‐organized for online delivery, but are not copy‐edited or typeset. Technical support issues arising from supporting information (other than missing files) should be addressed to the authors.

Supplementary
