# Supplementary material for: Coupling Infusion and Gyration for the Nanoscale Assembly of Functional Polymer Nanofibers Integrated with Genetically Engineered Proteins
Source: Macromol Rapid Commun. 2015 Jun 1;36(14):1322–8. doi: 10.1002/marc.201500174 (PMC5215549; doi:10.1002/marc.201500174)
Supplement: Supplementary file 1 — Supplementary [file MARC-36-1322-s001.pdf]

Copyright WILEY-VCH Verlag GmbH & Co. KGaA, 69469 Weinheim, Germany, 2015.

Macromolecular Journals

Supporting Information

for *Macromol. Rapid Commun.*, DOI: 10.1002/marc.201500174

Coupling Infusion and Gyration for the Nanoscale Assembly of Functional Polymer Nanofibers Integrated with Genetically Engineered Proteins

Siqi Zhang, Banu Taktak Karaca, Sarah Kay VanOosten, Esra Yuca, Suntharavathanan Mahalingam, Mohan Edirisinghe,* Candan Tamerler*

Supporting Information

Coupling infusion and gyration for the nanoscale assembly of functional polymer nanofibers integrated with genetically engineered proteins

Siqi Zhang[1], Banu Taktak Karaca[2], Sarah Kay VanOosten[2], Esra Yuca[2], Suntharavathanan Mahalingam[1], Mohan Edirisinghe[1]*, Candan Tamerler[2]**

[1]Department of Mechanical Engineering, University College London, Torrington Place, London, WC1E 7JE, UK

Email: m.edirisinghe@ucl.edu.uk

[2]Bioengineering Research Center (BERC), Department of Mechanical Engineering, University of Kansas (KU), Lawrence, KS 66045, USA.

Email: ctamerler@ku.edu

Expression and Purification of Red Fluorescent Gold Binding Fusion Proteins

The pDsRed-Monomer expression vector (Clontech) was used for the cloning and expression of the *dsred* gene in *Escherichia Coli 2507 (E. coli 2507) cells*. Both the coding sequence of DsRed as well as the Au-BP2 peptide was inserted into the pMal-c4x vector (NEB) to obtain MBP-DsRed-AuBP2 protein. The cells harboring the expression vector were cultured in LB (Luria-Bertani) medium (10 g/L tryptone, 5 g/L yeast extract, and 5 g/L NaCl, pH 7.0) supplemented with 100 µg/ml ampicillin. The cells were grown at 37 °C to an optical density of 0.6 at 600 nm at which point protein induction was initiated by the addition of IPTG (0.4 mM) and cells were incubated for 48 h.

Following the expression of protein, cells were harvested by centrifugation at 4000(×g) for 30 minutes at 4°C. The centrifuged cell pellet was re-suspended in column buffer (20 mMTris-HCl, 200 mMNaCl, 1 mM EDTA, pH 7.4) and disrupted by sonication at 200 W for 1.5 min (pulsed on/off 10 s each) using Branson Digital Sonifier with a double stepped micro-tip. Cell debris was removed by centrifugation (15000xg for 30 min at 4°C) and the supernatant was collected. The supernatant was sterile filtered as crude extract and 10 mL was loaded onto an amylose resin column (2.0 x 15 cm) equilibrated with column buffer. Following the removal of the unbound proteins, MBP-DsRed-AuBP2 fusion protein was eluted from the column with elution buffer (20 mMTris-HCl, 200 mMNaCl, 1 mM EDTA, 10 mM Maltose, pH 7.4). Next, the MBP tag was cleaved and removed (detailed procedure given below). The fusion protein was analyzed by SDS-PAGE (sodium dodecyl sulphate polyacrylamide gel electrophoresis) on a 4 % stacking gel and 12 % separating gel. Gels were stained with Expedeon Instant Blue solution for 15 min at ambient temperature (~20°C) and destained with DI water for 10 min.

The fusion of the gold binding peptide, AuBP2, to the full-length DsRed-Monomer protein was accomplished using complete gene synthesis. A Maltose Binding Protein (MBP) tag was used to facilitate protein purification. To construct the fusion proteins and expression vector, an exo-proteolytic restriction site was localized between the malE gene and the N-terminus of the fusion protein for subsequent cleavage using Factor Xa protease enzyme. The desired vector was created by designing oligonucleotides and their primers based upon the known AuBP2 peptide sequence, "CGP-WALRRSIRRQSY-GPC", with the addition of a spacer sequence, "SGGG", inserted between the peptide and the DsRed-Monomer on the expression vector (Supporting Information, Figure S1). DsRed-AuBP2 encoding gene was cloned into the expression vector, pMALc-4X, harboring malE gene. Next, the pMALc-4X-DsRed-AuBP2 plasmid was transformed into *E. coli* 2507. The transformed cells were used to express and purify the multifunctional proteins using an MBP-tag which was later cleaved from the DsRed proteins by Factor Xa proteolytic enzyme. Further purification was accomplished using copper chelating affinity chromatography to remove the cleaved MBP tag and DsRed proteins (Supporting Information, Figure S1). Purified proteins yielded a maximum red fluorescence when the protein was excited at 556 nm which follows the expected excitation and emission maxima of the DsRed-Monomer at 556 nm and 583 nm, respectively. The gold binding affinity of the fusion protein was also confirmed through AFM analysis carried out on a gold surface (Supporting Information, Figure S2).

The engineered fusion protein contains two functional domains, specifically, red fluorescent protein (DsRed) and a highly specific AuBP2c peptide tag (Supporting Information, Figure S1a). The pMALc-4X expression vector that encoded themaltose binding protein (MBP) was chosen due to its ease of cloning, over-expression, and purification. The DsRed-AuBP2 gene was cloned

into the pMALc-4X expression vector and transformed into an expression host cell, *Escherichia coli* (ER2507; Supporting Information, Figure S1b). Positively identified clones were induced 0.3 mMIPTG for 48 h to encourage over-expression of the DsRed-AuBP2 protein at 30°C. We purified the expressed proteins by applying two different column chromatography techniques. First, MBP-tagged affinity chromatography as used to purify MBP-DsRed-AuBP2. Pure protein fractions containing MBP-DsRed-AuBP2 were concentrated to 5 mg/ml of volume by 10000 MWCO of Amicon Ultra-15protein centrifugal filter (Merck Millipore, USA). Then, protein samples were transferred into 1X cleavage buffer containing 20 mMTris-HCl, 100 mMNaCl, 2 mM $CaCl_2$ (pH 8.0) by ultrafiltration using the same centrifugal filter tube. 40 µl of 1 mg/ml Factor Xa (New England Biolabs, USA) was added to 2.5 mg/ml of fusion protein in 1X cleavage buffer. The cleavage reaction was performed overnight at 16°C. The second affinity chromatography technique was used for further purification of the DsRed-AuBP2 bifunctional protein. The red fluorescent protein, DsRed, has a unique binding affinity for copper ions, therefore a copper chelating affinity

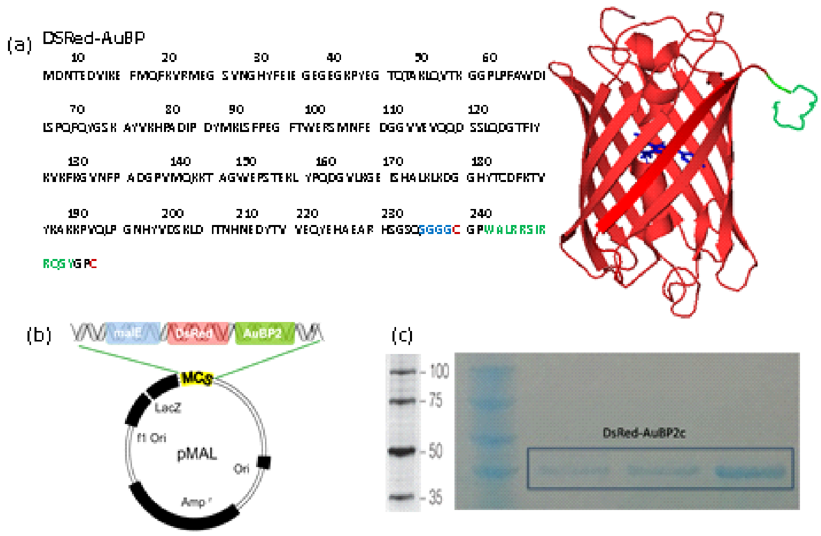

Supporting Information, Figure S1. Vector Design and Expression of DsRed-AUBP2c protein. Functional domains of the engineered protein, DsRed protein combined with gold binding peptide through a spacer (a), Schematic of vector construction (b), SDS-PAGE result of DsRed-AuBP2c (~30kDa) following the affinity column purification (c).

chromatography was utilized for the final purification step. On the basis of this interaction, the purification method was performed using copper immobilized column to remove unbound MBP-tagsas well as Factor Xa enzymes following the cleavage reaction. As the crude protein was passed through the copper immobilized column, DsRed-AuBP2 bound to the resin whereas other interfering proteins did not bind to the column and hence were removed. DsRed-AuBP2 was eluted using a competitive ligand imidazole that binds strongly to copper ions within the column, displacing our purified protein. The purity of DsRed-AuBP2 was analyzed by SDS-PAGE and gave a single sharp band around ~30kDa for DsRed-AuBP2 (Supporting Information, Figure S1c).

Self-assembly of DsRed-AuBP2c on Au substrate

In previously published work, we have extensively studied combinatorial-selected and bioinformatically-optimized peptides and have described their adsorption properties and role in surface binding.[12,13,16] In the current work, we studied the assembly of the fusion protein onto a gold surface. First, we generated a gold surface using micro-contact printing (μCP) and applied the proteins directly onto the surface. An AFM image (Supporting Information, Figure S2b) was recorded in tapping mode with a silicon cantilever at a 1 Hz scan rate and shows that DsRed-AuBP2c strongly binds exclusively to the gold region of the patterned surface. The AuBP2c peptide tag was confirmed to provide self-immobilization on the gold surface with consequential direction for the engineered fluorescence protein to self-assemble and develop a protein-based pattern.

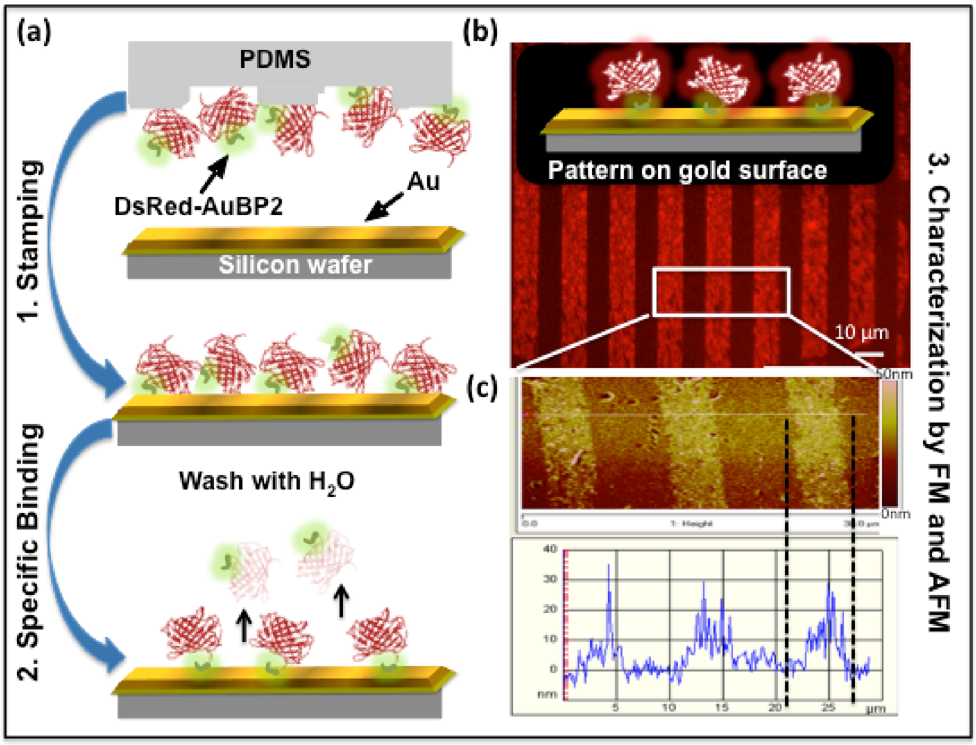

Supporting Information, Figure S2: Schematic representation of the generation of two-dimensional arrays of immobilized proteins on a patterned substrate fabricated through µCP (a) and fluorescence microscopy (FM) image of gold substrate following self-assembly of DsRed-AuBP2c (b) AFM image of gold substrate following self-assembly of DsRed-AuBP2c with height profile of the arrays (c)

Engineered protein coated AuNP attachment on the nanofibers

A PEO-water system was used to generate the fibers. The DsRed-AuBP2 protein was incorporated through the addition of functionalized AuNPs. Supporting Information Figure S3 shows the attachment of protein-AuNP mixture dispersed in the fibers. In addition, Supporting Information, Figure S4 shows the electron microscope verification of AuNPs attachment to the fibers.

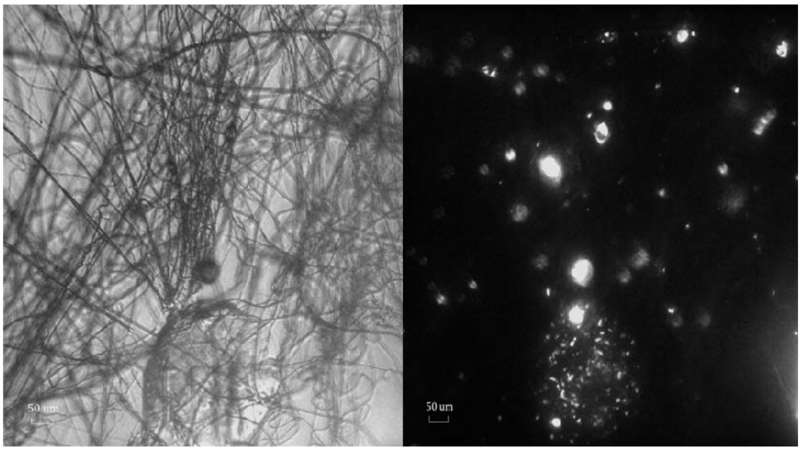

Supporting Information, Figure S3: Ds-Red-AuBP2 proteins attached to the fibers. Left side shows bright field image and the right side shows the corresponding fluorescent image.

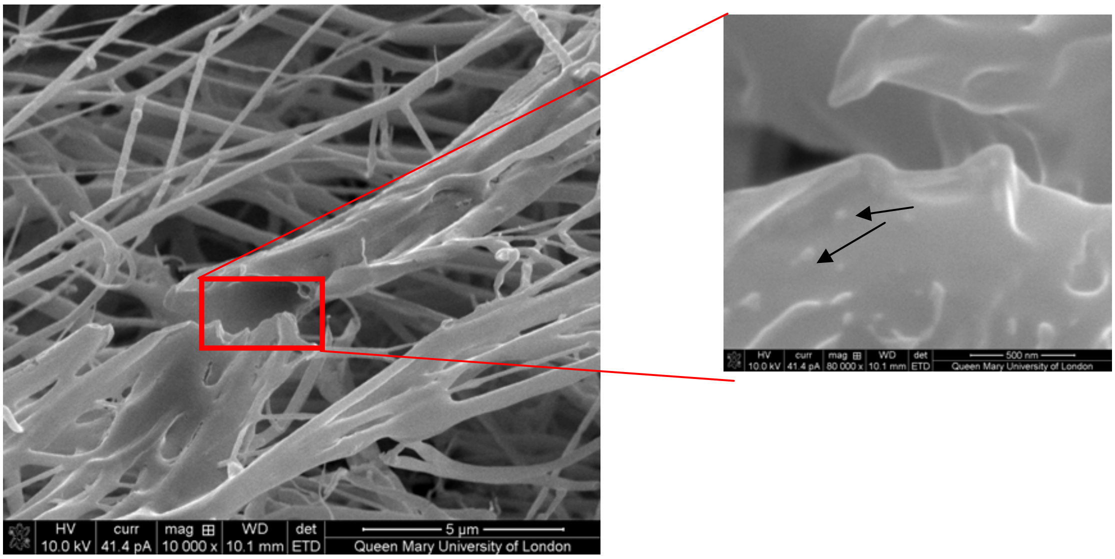

Supporting Information, Figure S4: AuNPs attachment to the fibers are verified using focused ion beam (FIB) – SEM using a dual beam (Helios) machine. The fibrous sample is cut through a section using low beam current (40 pA) and the dispersion of NPs on the surface (indicated by arrows) was observed.

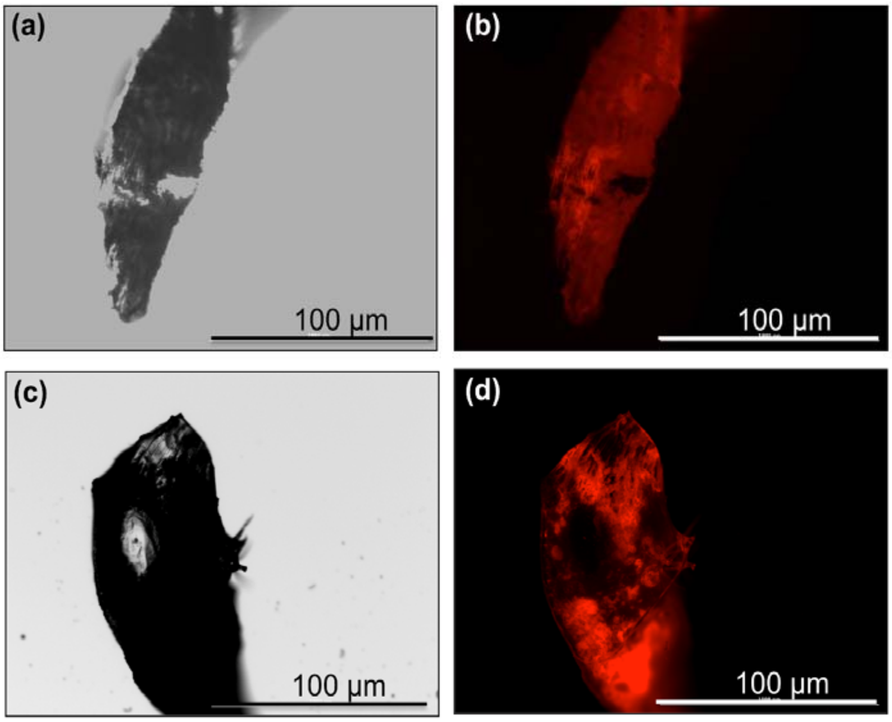

Supporting Information, Figure S5: Fluorescence Microscope image of protein integrated nanofibers (a-b) samples were in PBS buffer, (c-d) samples in dry condition.
